# Objectifying “Pain” in the Modern Neurosciences: A Historical Account of the Visualization Technologies Used in the Development of an “Algesiogenic Pathology”, 1850 to 2000

**DOI:** 10.3390/brainsci5040521

**Published:** 2015-11-17

**Authors:** Frank W. Stahnisch

**Affiliations:** Department of Community Health Sciences & Department of History, The University of Calgary, 3280 Hospital Drive NW, Calgary T2N 4Z6, AB, Canada; E-Mail: fwstahni@ucalgary.ca; Tel.: +1-403-210-6290

**Keywords:** pain, history of medicine, Leipzig, Montreal, New York, nineteenth century, precursors to functional neuroimaging of pain, twentieth century

## Abstract

Particularly with the fundamental works of the Leipzig school of experimental psychophysiology (between the 1850s and 1880s), the modern neurosciences witnessed an increasing interest in attempts to objectify “pain” as a bodily signal and physiological value. This development has led to refined psychological test repertoires and new clinical measurement techniques, which became progressively paired with imaging approaches and sophisticated theories about neuropathological pain etiology. With the advent of electroencephalography since the middle of the 20th century, and through the use of brain stimulation technologies and modern neuroimaging, the chosen scientific route towards an ever more refined “objectification” of pain phenomena took firm root in Western medicine. This article provides a broad overview of landmark events and key imaging technologies, which represent the long developmental path of a field that could be called “algesiogenic pathology.”

“The past of a present-day science is not the same thing as that science in the past.”

(Georges Canguilhem) [1]

## 1. Introduction

This article considers several noteworthy trends in the history of psychophysiological pain research and neuroimaging techniques as these became used in this particular field from the nineteenth century onwards. Already during classical antiquity, “pain” had been widely understood as an unpleasant sensory and emotional phenomenon, yet cultural responses to pain were likewise very heterogeneous and could not be seen as clear-cut or evenly culturally recognized. The classical perceptions of pain themselves had varied with differing social living conditions, with patients’ nutritional status, along with their respective spiritual and religious behaviors. With the advent of what could be called an “algesiogenic pathology” during the nineteenth century, nonetheless, the increasing use of experimental and scientific research techniques became a signifier for the modern conceptualization of pain and pain-related conditions. Pain now emanated as the subject of objectification, measurement, and eventually medical visualization by way of functional neuroimaging techniques. In this sense, the precursor techniques in pain visualization were based on animal experimentation and human studies. They helped to investigate the complex connections of individual and often exclusive sensory, emotional, and integrative actions of the human nervous system. This endeavor should help in the elucidation of the cognitive and emotional components of the widely prevailing “pain” phenomena from a neuroscientific perspective [2].

Throughout the century before the last, a major shift in pain research had taken place, demonstrated by the employment of more subjectivist accounts [3] within ever increasing objectification attempts of pain that were based on experimental evidence accounts [4]. This shift became particularly associated with the introduction of innovative experimental pain concepts, such as the notion of “threshold values” for the elucidation of pain perceptions or the establishment of the scientific “normal values” in pain parameters. The latter likewise regarded the scope, quantity, and intensity of pain as it could then be measured in the physiological laboratories of the time [5]. Especially, the investigations in psychophysiological pain research by Johannes Mueller (1801–1858) [6] and Maximilian Ruppert Franz von Frey (1852–1932) [7] in Germany (during a time period of almost fifty years from 1840 to 1890), led to intriguingly new foundations in this field of the modern neurosciences. Since the middle of the nineteenth century, neurophysiological researchers, such as Theodor Fechner (1801–1887) in Leipzig [4], Wilhelm Wundt (1832–1920) in Heidelberg and Leipzig [8], or von Frey in Wuerzburg and Zurich, started to analyze the causes [9], the spread, and individual perceptions of “pain” in the nervous system through systematic experimental investigations. Theodor Fechner’s seminal publications made the contemporary neurophysiologists aware of the potential inclusion of psychological and subjective perceptions as respectable objects for study in both laboratory settings and clinical wards. At the same time, Wundt frequently crossed the boundaries between animal and human subject research, which opened novel theoretical considerations incorporating pluridisciplinary perspectives for the continuing development of experimental pain research. On the experimental side, Wundt had himself worked with many physiological methods that included innovative visualization techniques as well [10]. He carved out intriguing theoretical psychophysiological considerations and provided detailed philosophical analyses of the new experimental findings, which often included the assessment of subjective accounts of pain perceptions in the respective test individuals. While each one of these neurophysiologists’ research programs has been studied in their own right (e.g., von Frey’s approach to clinical diagnostic examination, as well as Wundt’s and Fechner’s scrutiny of the psychophysiological laws that govern pain perception), their contributions to an emerging field of biomedical, psychophysiological, and philosophical pain studies have so far not been sufficiently historically contextualized [11]. This likewise pertains to their highly sophisticated instruments and apparatuses, which they applied to the study of pain physiology, most notably in the case of von Frey on his medical wards at several universities in southern Germany. The resulting pain-measurement devices became also employed for diagnostic purposes in many patients with acute or chronic pain disorders towards the *Fin de Siècle*.

In certain ways, the respective time lag between early psychophysiological research and the application of such findings in contemporary medical clinics could also be explained as a process towards the redefinition of the boundaries of experimental instrumentation. This process situated the physiological apparatuses, which had likewise transformed the spectrum from threshold values to normal values [12], and it now also led to the recalibration and balancing out of such values in the new field of pain investigations. Until today, such elements as the phenomenological “identification,” “evaluation,” and “physical reduction” of pain, which these pioneers had put on the scientific map of nineteenth century medicine, supplement the endeavors and clinical repertoires of modern pain research [13]. In the history and theory of modern neuroscience, recourses to subjective forms of reasoning and creative experimental practices did not just follow the expectation of materialistic and reductionist physiologists, but also reflected the autonomous effects of observation, instrumentation, and measurement results. Physiologists, such as the Berlin group of “physical physiologists,” comprising Emil Du Bois-Reymond (1818–1896), Ernst von Bruecke (1819–1892), and Hermann von Helmholtz (1821–1894), envisaged neurophysiology as gaining more diagnostic certainty if it tried to increase the physical autonomy of the measuring instruments and apparatuses. The nineteenth century thereby marked a decisive period in the history of medicine, during which the neurophysiological and animal experiments of Mueller, Wundt and von Frey built the foundations for a new experimental tradition in modern pain research [14]. From the German-speaking countries, these new pain research trends soon spilled over into the medical and scientific communities of several other countries, including France, Britain, and the United States.

At the same time, neurologists were clearly fascinated by viewing such images of the body that displayed intriguing disorders, which could be understood as typical for a broader array of clinical symptomatologies. Such neurophysiological images, for example comprised by kymographic read-outs, skin-plethysmographic descriptions or early patient photographs, were analyzed in the relative privacy of the laboratory or in medical professional meetings. Standardized motion studies were viewed ever more closely and came to be experimentally reproduced, whether through the loop projection of films at scientific gatherings or through frame analyses, while these were consecutively published in professional and scientific journals [15]. Subjective states of pain and distress, as described in the accounts of individual patients, were no exception from that rule. Yet, what was the neurologist’s stake in subjecting their patients to this kind of optical scrutiny? Many of the source data, which were considered in neurological settings for example, also testified to the challenging conditions of patients’ intense somatic and psychical pain [16]. Patients’ agency became a crucial issue in the process of a new medical form of documentation; it thereby also drastically reduced the person imaged to the “signs” and “symptoms” of physiological identification [17]. One could thus suggest that the patients, who were included in the experimental protocols of the nineteenth century, morphed into subjects of a new way of “neurological seeing” by their doctors. It brought the methodological issue of the relation of “pain” and “angst” ever closer together for the field of phenomenology and physiology [18].

There are furthermore decisive historical connections between pain measurement practices and the conceptual processes by which pain had been understood. These processes were based on an emerging notion of pain as a measurable, “normalizing” and, thus, objectifiable phenomenon. It often phenomenologically resisted the very same experimental tendencies that were also present in the ongoing debates of the preceding eighteenth century [19]. Clearly, efforts at pain measurement also furbished new theoretical understandings of the neurophysiology of the nervous system. And these research developments visibly contributed to the continuing processes of pain research after World War Two. The respective working definitions of the measurement and conceptualization of pain, however, can be seen as central for the objective of this article, when referring to the scientific attempts in the identification and characterization of pain thresholds, tolerance breadths or psychological reactions to analgesics. Experimental measurement by this means grew in importance, especially in the respective development of new basic and clinical experimental protocols. It sought to characterize variability in pain attributes among individuals and groups, as well as the neurophysiological mechanisms that underlie such attributes [20]. The objectification of pain further characterized a strong shift in the prevailing understanding and experience of pain among patients and clinicians. Around the middle of the nineteenth century, the medical and cultural concepts of pain converged in a uniform and largely physiologically informed stimulus-process that contributed to the new interpretation of reflex actions within the nervous system. The stimulus-process encompassed an activation of peripheral nerves by noxious stimuli, the movement of pain signals in fixed pathways, or their effects for a cerebral location “registering” the pain. However, by the end of the century before the last, the notion of a “noxious stimulus” had become further refined. It was augmented through a new way of thinking, which regarded pain as experimentally measurable in laboratory settings. Conversely, such experimental settings often enough marginalized the individual experiences of the test persons, as well as the social meanings that they ascribed to their perception of “being in pain” [21].

The current article for that reason explores how investigations of pain since the experimental protocols of the nineteenth century had entered into a state of pain’s laboratory objectification. When the history of neurophysiology and neuroscience is taken into account, neurophysiological research in the nineteenth century furthered the understanding of the pathways and pathological conditions of pain in ever more detail. Neurophysiology suggested how such subjective factors as the patients’ personal history could be understood in terms of the nervous system’s integration of pain sensations. Moreover, this trend confirmed a uniformity of answers to specific experimental stimuli in both inter-subjective settings and also between individual histories of patients’ pain experience [22]. In the first part of this article, the history of pain measurement shall be mapped and the tendency towards quantifying pain since the middle of the nineteenth century worked out. The second part follows this tendency into the twentieth century and explores the trends in scientific pain objectification from physiological and pharmacological perspectives to the work of American anesthesiologist Henry K. Beecher (1904–1976). In the third part of the article, the evolution of the McGill Pain Questionnaire (MPQ) shall be reviewed. The MPQ represented a pain assessment tool that was based on the integration of measurement and visualization protocols of “pain” during the first postwar decades. The conclusion ties such examples to the insightful connections between neurophysiological measurement and the subjective patient experiences, which became prevalent throughout the modern history of pain [23].

## 2. Brief Overview of the History of Experimental Pain Research

In 1989, the American anesthesiologist Dennis Turk remarked that “the investigator trying to measure pain is in the position of the hunter who goes into the woods to find an animal no one has ever seen” [24]. At the bottom of this statement manifestly lay the results of research observations made by thousands of basic scientists and clinicians who had constructed, designed, and adjusted experimental methods, scales, and observational methods for the quantification of pain. When this research development is hard pressed against an epistemological backdrop of the more technology-based pain research programs, one could venture so far as to describe the history of pain measurement as a scientific “failure”, as several historians have done in the past [25]. We need to acknowledge that the measurement units, assessment tools, and psychophysiological scales can hardly be regarded as uniform. They changed repeatedly over time, while the differences in the social and cultural acceptance of pain and distress were often neglected in quantitative studies of subjective responses [26]. The applied research instruments and apparatuses, that are otherwise so powerful in identifying often-minimal changes in physiological organ functions, remained comparatively numb for the delineation of the psychosomatic intricacies of human pain. Since the time of the nineteenth century, however, attempts to quantify and depict pain have generally furthered neuroscientific knowledge about the role of nerve fibers, the pain pathways, and the integrative actions of the nervous system at large. This research tradition likewise pondered how external factors, such as the history, behaviors, and circumstances of the patients, could influence neurophysiological pain processes [27].

The historical attempts to objectify “pain” may be separated into three distinct approaches: psychophysiological experimentation in the laboratory, the use of standardized questionnaires, and the measurement approaches of clinical rating scales. With the second half of the nineteenth century, experimental psychophysiology created new apparatuses, practices, and visualization strategies for the isolation and measurement of pain, while contextualizing the laboratory stimuli in line with the test persons’ responses. German experimental psychologist Fechner from the University of Leipzig attempted to establish a mathematical law that governed stimulus intensity and perceptions resulting from the representation of “the mathematical form of the link between a physical stimulus and our perception of it,” Fechner proposed a method of scaling sensations based on “just noticeable differences;” he also recorded the objective magnitude of just noticeable differences at different levels of the stimulus [4]. Seventy years after Fechner had published his *Elemente der Psychophysik* (1860), his concepts would still influence the design of pain measurements by surgeon James D. Hardy (1918–2003), neurologist Harold G. Wolff (1898–1962) and research associate Helen Goodell (1920–2004?) at Cornell University [28]. The psychophysiological experimentation of the nineteenth century built the foundation of many later research trends to incorporate subjective accounts for the measurement of health and disease as well. Ian McDowell from the University of Ottawa’s Community Health Sciences has stated that “psychophysics is concerned with the way in which people perceive and make judgments about physical phenomena such as the length of a line, the loudness of a sound, or the intensity of a pain: psychophysics investigates the characteristics of the human being as a measuring instrument” [29]. This tradition has even held on to the reception of the MPQ as a foundational questionnaire, which instigated many trends in modern clinical pain research since the twentieth century [30]. Furthermore, the emergence of modern neuroimaging technologies has been greatly influenced in this way, as a refined and objectifying approach to measuring threshold values, the tolerance breadth, and the development of chronic pain features into one encompassing theory [31].

## 3. The Leipzig School of Psychophysiology

**Figure 1:**
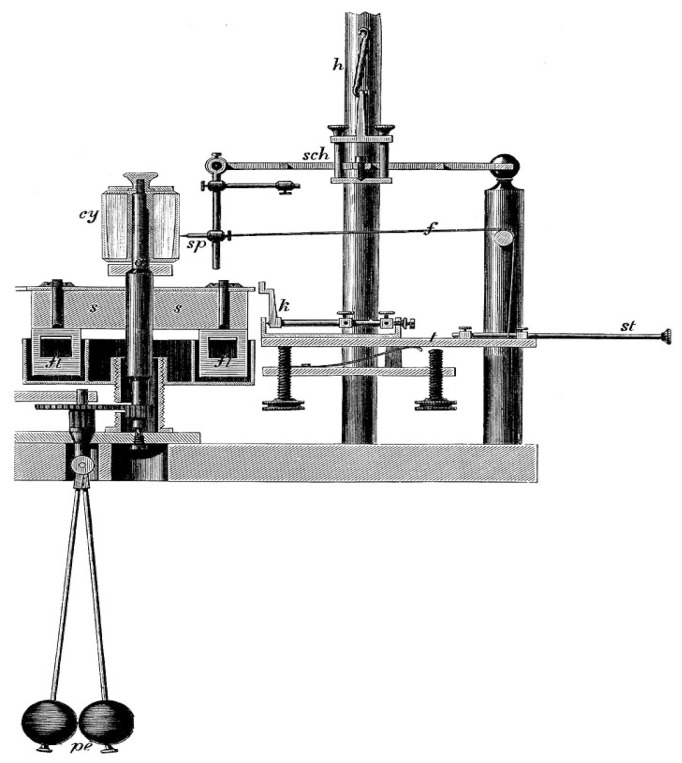
Hermann von Helmholtz’ myographion for the experimental determination of pain conduction and reaction time measurement (1891) [35].

While the tradition of an objectification of pain visibly started with the early experimental approaches introduced by the German physiologist and physicist Hermann von Helmholtz [32], it needed almost two decades for a full application of such experimental protocols in the field of perception and sensory physiology as well. Among the important foundations that Helmholtz had contributed to the field further count the determination of the temporal courses involved in perceptual processes, along both the peripheral and central nerve tracts. A primary example for him was the elucidation of the auditory processes between the sensory organ of the ear and the human auditory cortex, in which Helmholtz interposed physical auditory processes with sensory ones. He thought that humans could not perceive “faster” than the actions of the nervous force (*Nervenkraft*) admitted [33]. Likewise, the transmission of pain and touch appeared as an instantaneous process that was elicited through a “sudden change in the nervous state” (*Nervenzustand*). In referring back to Johannes Mueller’s “*Law of Specific Sense Energy*”, Helmholtz postulated the general translatability of external sensory stimuli into corresponding nervous answers, which he investigated from a qualitative rather than a measurement perspective (Figure 1) [34].

While Helmholtz widely argued against the possibility of an introspective form of psychology that could use subjective input for the use of laboratory experimentation, such as test persons’ descriptions of pain sensations as well as their duration and quality, there already existed a considerable research community outside borders of the materialist tradition of the Berlin group of physical physiologists [36]. This trend was particularly associated with the emerging school of psychophysiologists at the University of Leipzig and its engagement with measurement approaches towards an “objectification” of pain. The school’s representatives expanded the experimental paradigm to include psychological phenomena into their laboratory studies as well [37], by aligning specific experimental investigations of test persons with physical and mathematical methods. The beginning of the tradition emanated especially with the publication of Ernst Heinrich Weber’s (1795–1878) book *De Tactu* (“*Ueber den Tastsinn*”) in the year 1834 [38]. Weber held the chair of anatomy and physiology at the University of Leipzig and had himself been introduced to experimental physiology by former pupils of Johannes Mueller in Berlin. The latter, in return, continued to receive Weber’s work on the localization of the anatomical pain fibers in the skin through the association of clinical patient observations with detailed morphological studies and animal observations:

“In those areas of the skin, in which a close association of the pain fibers in the stimulation experiment can be assumed, Weber has also made associated observations. According to him, the differences in applied temperatures and weights have likewise been very adequately been discriminated. Even increasingly larger weights could be described by the test persons, when they had been applied to those areas of the volar side of the fingers, for example, other than being applied to the skin at the front of the head.” [6].

**Figure 2:**
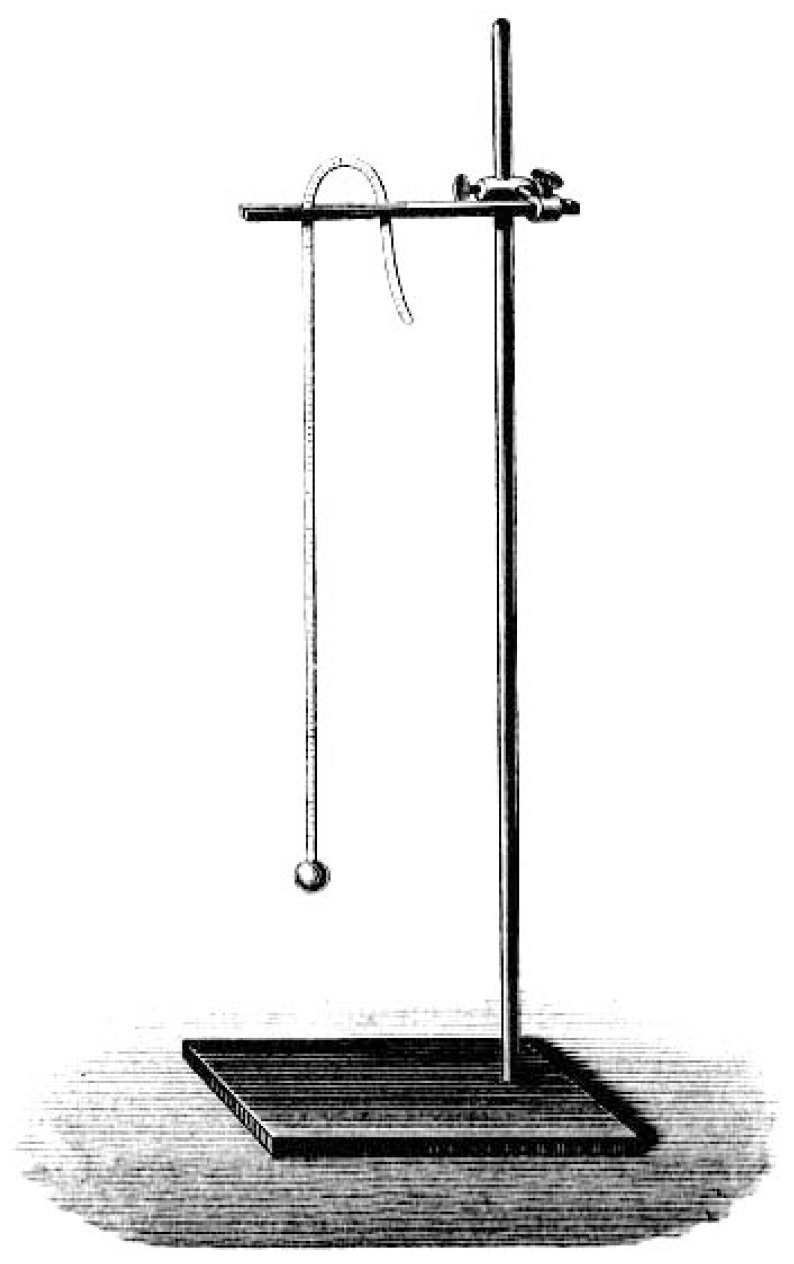
Heinrich Weber’s metronome (1834), as it was used for time measurement of pain and touch phenomena in human test persons [41].

It is remarkable that Ernst Heinrich Weber was only able to develop his morphological hypotheses on sensory organ structure, after he had gained direct access to the body phenomenology in his test persons. For the purposes of the discrimination of weights, sound levels, warmth, *etc*., he began to classify the research examples according to their intensity, scope, and the quantity of the sensory perceptions in the respective research program. In his quite precise findings, Weber could for example show that the difference between individual weights could be told until a threshold of 1/30 of the original weight was surpassed, had the test persons been given different sets of cylindrical metal weights into their hands, for which Weber designed intriguing logarithmic tables to visualize the differences, a protocol that he later also extended to pain research [39]. However, Weber’s intriguing psychophysiological findings became only introduced in an indirect form into the medical and physiological text books of the time, although Johannes Mueller, Carl Friedrich Wilhelm Ludwig (1816–1895) and, later, Ludimar Hermann (1838–1914) had received them early on. Instead, the important “subjective” dimension of the experimental protocols remained left out of these later descriptions. Such tendencies towards objectifying the sensory perceptions in the experimental test outcomes and visualizing the resulting data in logarithmic tables nevertheless broke with Weber’s original conceptualization. In fact, he had repeatedly stated that the test persons’ self-descriptions marked an important entry point into the experimental measurement protocols (Figure 2); and this went noticeably beyond the idea of an individual stimulation threshold [40].

It can be easily assumed that the main protagonist of experimental psychophysiology in Germany at the time, the physicist Gustav Theodor Fechner, could build on the previous works of his colleague at the University of Leipzig [9]. Like Weber, Fechner combined psychological questions with precise physical and mathematical methods, researched the threshold values and norm values of the investigated perceptions, and tried to elicit the relationship of stimulus intensity and sensory perceptions in various psychological processes. In his book “*Elements of Psychophysics*” (1860), Fechner gave an encompassing overview on a still blossoming research tradition, which nevertheless had the potential to develop into:

“(…) an exact science of the mind and brain relationship. As such an exact science, psychophysics could draw on both the physical experiences as well as the mathematical relationships between perceptual facts. The latter would necessitate that a particular state of perceptual facts was incorporated to inform the research progress. After the scope of the mental faculties had been explored in preliminary research, it could be seen as the book’s primary task to determine the quantity of the specific mental faculties further and to combine it with a second level of applications and uses which would ensue from the already established research thus far.”

[4]

It is also possible to interpret Fechner’s remarks in his seminal book as an attempt to delineate the borders of the field of psychophysiology *vis*-*à*-*vis* the earlier tradition established by Mueller and von Helmholtz that had primarily concentrated on the physiological determination of the anatomical and functional processes in the sensory organs and nervous system itself. Physical physiology, for him, had ignored the perceptual and behavioral aspects of human psychophysiology. Fechner thus attempted to reach at a much more extensive program, when he wrote in a notable publication of 1851 on the “Conceptions of Psychophysiological Parallelism”. In this work he claimed that all mental processes would have an exclusive subjective dimension, while bodily processes fell both within subjective as well as objective dimensions (*Koerperliches*, *Leibliches hingegen als solches vermag immer nur einem anderen als sich selbst zu erscheinen*), which would hence become experimentally accessible to the physiologist [42].

**Figure 3:**
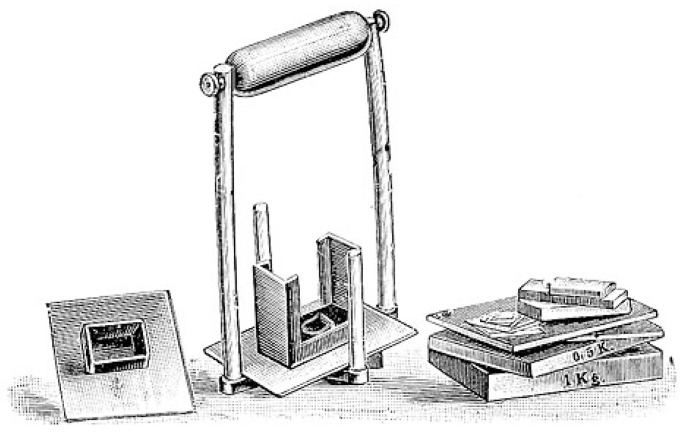
Gustav Theodor Fechner’s experimental weights to calibrate subjective perceptions in his test persons (1856) [44].

**Figure 4:**
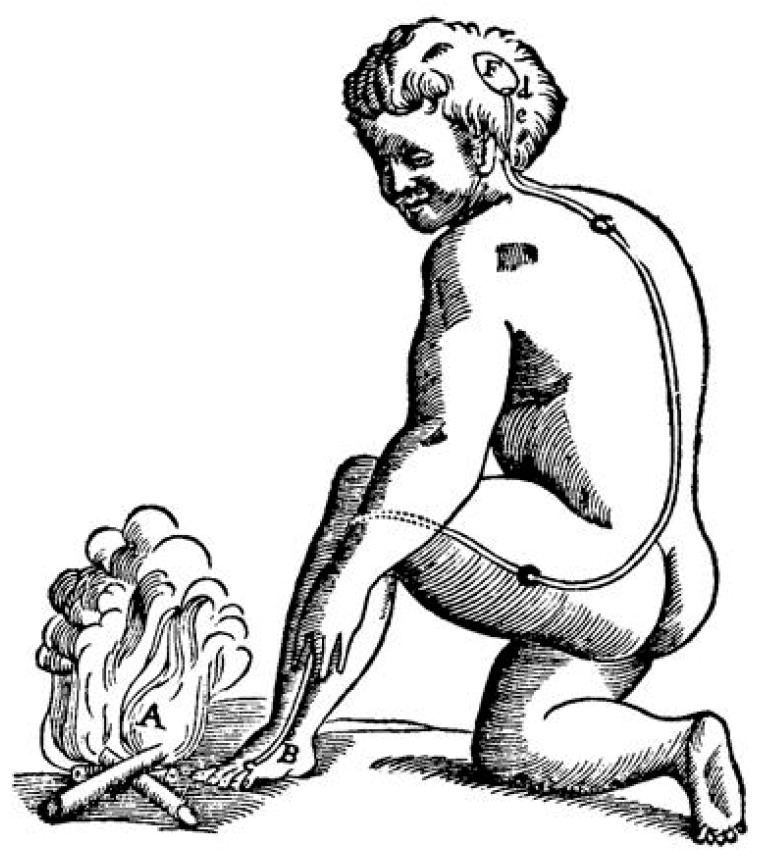
Descartes’ depiction of nerve transmission in a man who perceived burning heat at an open fire through the sensory nerves of his toe (1664) [45].

Fechner’s groundbreaking work also offered important new insights into the development of a new scientific “algesiogenic pathology”, in that it introduced a very detailed notion of “threshold values” into the psychophysiological program of experimental pain research. Decisive in this respect was Fechner’s assumption of particular consciousness levels that interacted in human sensory perception in quite complicated though intricate ways (Figure 3). The perception of pain became coupled with local physiological process in the skin and abdominal organs, *etc*. (*Schmerzbewusstsein*), while the full dimension of pain differences and the phenomenon of chronic pain could only be conceived in line with the main cognitive processes of the mental faculties (*Hauptbewusstsein*). This differentiation should make it possible for him to explain the discontinuity of several subordinated perceptual levels in human sensory neurophysiology. Fechner’s primary example in this respect was the motor reflex action that was elicited through the original pain perception, even though no cognitive involvement had taken place (e.g., the sudden withdrawal of the hand from a hot stove or fire), as it had been classically described by Réné Descartes (1596–1650) (Figure 4) [43].

**Figure 5:**
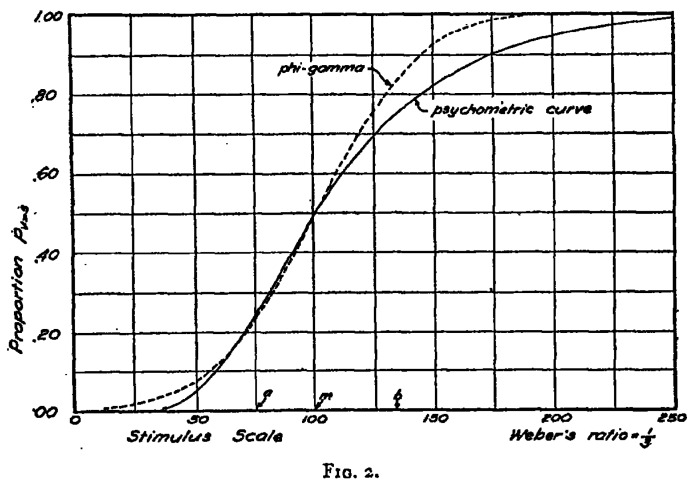
Gustav Theodor Fechner’s logarithmic tables of sensory isomorphisms (1858) [47].

According to Fechner, neuronal excitations supported the nerve processes in other parts of the human nervous system through the crossing of nerve tracts until “sufficient nerve force” was reached, in order to trigger a conscious human impression. If sufficiently researched in detailed psychophysiological programs, Fechner thought it possible that the specific nervous conduction could be understood as a discrete stimulus values. In his experimental perspective, he even attempted a fully quantitative investigation of the stimulus-perception relationship, which had previously not been pursued. At the same time, Fechner also related to Weber’s definition of the “perception thresholds” of 1846 [40]. The impact that both Weber and Fechner exerted on the ensuing development of the field of experimental psychophysiology and neurological pain research can hardly be overestimated. Not only did they accentuate the importance of subjective self-descriptions and qualitative assessments of sensory perceptions, such as pain, in the first place; yet they also connected their findings in important ways towards an increasing standardization and normalization of the experimental findings among larger cohorts. This is profoundly visible in their development of logarithmic curves that were based on their “theory of isomorphisms” (Figure 5). As experimental psychophysiologists, Weber and Fechner let themselves be guided through their measurement series and they tried to circumscribe particular value areas that would allow for a determination of “normal” perceptive qualities. Based on these findings, Fechner introduced his foundational “logarithmic formula of perceptions” that provided orienting curves allowing for assessments of the mean distributions of sensory perceptions, such as the senses of temperature and pain [46].

**Figure 6:**
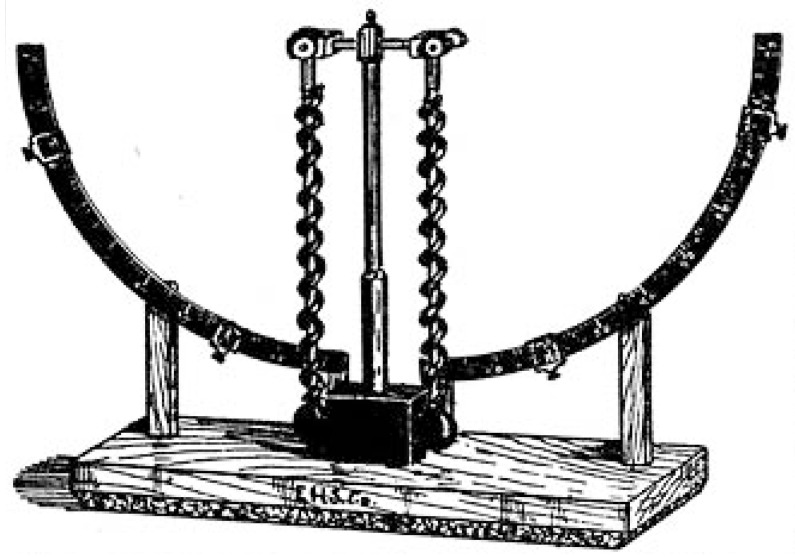
Gustav Fechner’s pendulum for psychophysiological time measurement in research with psychological test persons (1858) [49].

The introduction of Fechner’s logarithmic tables laid the basis for Weber’s later development of the so-called measuring formula (*Massformel*) (de = c × dr/r) that was included in his fundamental formula to describe the discriminatory qualities between individual personal pain perceptions. The variable of c was a non-yet-known constant that should still be more closely determined through the ongoing psychophysiological research programs. Based on Weber’s previous work, Fechner eventually conceptualized his psychophysical measurement formula, which was based on a mathematical integration process in a logarithmic form: E = k × (log *r* − log *r*0). In Fechner’s interpretation, k was a constant and *r*0 the value, which the stimulus *r* assumed, once the perceptual quality reached a certain intensity e = 0; *r*0 became thereby the threshold value of the stimulus *t*. With his mathematical analysis, Fechner contributed a new quantitative method to examine the physiological stimulus values in relation to the respective perception intensity during specific time intervals (Figure 6). Hence, the law was primarily used with significant practical relevance for sensory physiological investigations, both in psychological rest persons, as well as in clinical pain patients in neurology, psychiatry, and internal medicine [48].

**Figure 7:**
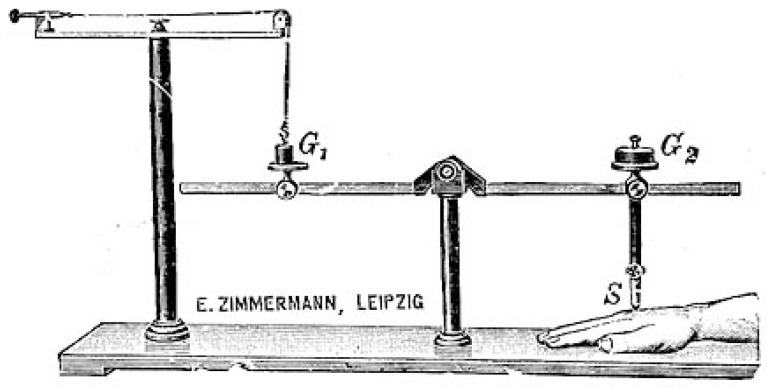
Wilhelm Wundt’s aesthesiometer was used for both scientific and educational demonstrative purposes, *ca*. 1883 [52].

A compromising position between the approaches of materialistic experimental physiology and experimental psychophysiology became eventually available through the Leipzig philosopher and psychophysiologist Wilhelm Maximilian Wundt. Other than Fechner, Wundt conceptualized in his work “*System of Philosophy*” of 1889 and the “*Foundations of Psychology*” of 1896 the theory of psychophysical parallelism epistemologically as a heuristic principle and not as a law of nature [50]. The subject of psychophysiology was for him not a dubious “mental substance,” but was the analysis of all causal relationships between mental and bodily processes. Wundt did not categorize any of the psychophysiological functions in a hierarchical way, yet he emphasized their parallel interactions in what he called the “chain of perceptions” (*Wahrnehmungskette*), including: nerve conduction → “apperception” → becoming conscious → vigilance → “will time” (volition) → motor activation. Wundt’s analyses of the “chain of perception” were centrally represented in the integrative approaches of the Leipzig school of physiologists and their pupils throughout Europe and North America (e.g., Carl Correns, 1864–1933, and John M. MacEachran, 1877–1971). Wundt’s chain of perception was based on introspective comparisons and the persistent correlation with “aesthesiometric” values (such as pain intensity, blood pleasure, temperature, *etc*.) (Figure 7). Eventually, the approach of the Leipzig school materialized in the foundation of a first Institute for Experimental Psychology in 1879, which became a hallmark in the general development of nineteenth-century psychophysiology of pain [51].

With the establishment of the Leipzig Institute (as the psychology historians Edwin Boring (1886–1968) and Horst Gundlach (b. 1944) have pointed out), the research programs of physiology and psychology went in separate disciplinary directions [53]. The vast majority of German-speaking universities had already fairly established (medical) physiology departments around the 1860s, while most of the psychology departments were only founded around the turn of the century, when the field of specialized pain research became further extended in the area of clinical medicine, as the example of von Frey at the University of Wuerzburg shows.

**Figure 8:**
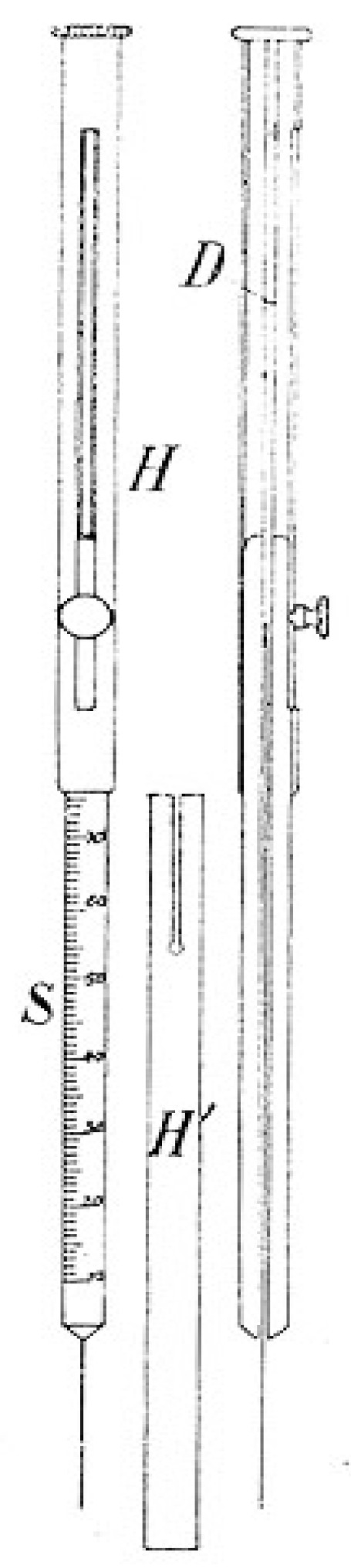
A basic hair aesthesiometer following Maximilian von Frey (1906) [59].

While many of the programs of the contemporary neurophysiological researchers have already been well examined, including the cases of Emil DuBois-Reymond in Berlin [54], Isidor Rosenthal (1836–1915) in Erlangen [55] or Ludimar Herrmann in Koenigsberg [56], their contributions to pain research remain rather under-reflected in the research literature. This likewise concerns their highly thoughtful and meticulously designed recording instruments and apparatuses, which particularly von Frey introduced into the clinical wards at the Universities of Wuerzburg and Zurich. As a neurologically interested physiologist, von Frey treated patients with both acute and chronic pain symptoms, for which he also designed specialized aesthesiometers (Figure 8). These instruments allowed for a better determination of hypo- and hyperaesthesia, prolonged pain symptoms, or the differentiation between neurological and psychiatric pain disorders. With the help of these measuring and visualizing techniques, von Frey thought to determine pain conditions in refined psychophysiological experimental series which focused both on the diagnostic and therapeutic aspects of scientific algesiogenic pathology [57].

The relatively long time delay between the early developments in physiological pain research and the introduction of these new approaches into the medical clinics of the latter half of the nineteenth century can also be described as an emancipation process of objectifying medical science, on the one hand, and the introduction of the new apparatuses into the clinical and research units, on the other hand. The experiments and apparatuses were thus regrouped on a spectrum from normal to threshold values and became research and visualization hallmarks of the new algesiogenic research field [58].

In this sense, the approaches developed by von Frey also impressed the early neurological surgeons Otfrid Foerster (1873–1941) in Breslau and Wilder Penfield (1891–1976) in Montreal. They likewise exerted a considerable influence on the director of the Clinical Neurology Department at the University of Heidelberg from 1920 to 1941, Victor von Weizsaecker (1886–1957). Von Weizsaecker even developed one of the first specialized academic psychosomatic wards (*Nervenabteilung*) in the German-speaking countries, which particularly included patients with pain syndromes that were both neurologically and psychiatrically treated [60]. It became a widely acknowledged and influential clinical division which included a range of holistic diagnostic and therapeutic approaches during the 1930s and 1940s. At the same time, von Weizsaecker developed his clinical understanding into a widened neuroscientific conception, by differentiating three different types of pain in his main publication “*On Pain*” (*Die Schmerzen*) of 1926. Similar tendencies can also be found in copious influential accounts in North America, as these emerged during the immediate postwar period: First, von Weizsaecker differentiated a cognitive and physiological side of an order of pain (*Schmerzordnung*) that was largely based on the experimental psychophysiological findings of the nineteenth century. Second, he included a developmental and morphological dimension of pain in his theoretical account, as it tried to incorporate the destructive pathologies found in cancer and neurodegenerative diseases, which he called “destructive pain” (*Zerstoerungsschmerz*). It was separate from a developmental form of pain (*Werdeschmerz*), as this for example emanated in skeletal growth processes in children during their early adolescent phase. Eventually, von Weizsaecker added a farsighted environmental dimension to the understanding of pain. Similar to the Leipzig school or the ecological views of Thure von Uexkuell (1908–2004), it interpreted the psychophysiological reactions as adaptation phenomena to external constrains—mediated between bodily phenomena in “pain”, “angst,” and “stress” [61].

A refinement of pain taxonomies occurred with the influential group of anesthesiologists in New York around Hardy, Wolff and Goodell, who designed new methods for objectifying pain measurement between the 1940s and 1950s. They intended to recognise biophysical constants in personal pain conditions, while their research focus centered on the mapping of early “sensations of pain” and patients’ prolonged interactions with it. For Hardy and his colleagues, pain could be experimentally measured, as long as it was localizable, diagnostically separate, and also treatable with specific analgesic drugs. For the New York group, a pain threshold could be defined as the “least perceptible intensity of pain,” while they sought to document their patients’ and test persons’ answers in a separately manufactured pain measurement scale [62]. In 1940, Hardy’s team reported on a new way of measuring pain thresholds, based on the design and use of a unique dolorimeter device. It was organized around a fine application of heat, rather than mechanical or electrical means, to elicit pain in the experimental setting. With the use of optical devices in the internal tube of the dolorimeter, it was now possible to project a light beam onto a specific spot on the skin of a test person by using relatively moderate heat energy levels for this procedure. Similar to Weber and Fechner in the mid-nineteenth century, Hardy and his coworkers wanted to elicit the smallest threshold necessary to lead to a pain session, an experimental paradigm that was soon widely received [63].

One such group stimulated by Hardy’s group was an Oregon-based team of scientists around the anesthesiologist Frederick Haugen (1908–1968?) and the surgeon William Livingston (1892–1966), who reported on their experiences with the dolorimeter. The Oregon group eventually found that the effects of the heat-dolorimeter were largely a function of use regularities along with the instrument’s operation time. Their experiments emphasized individually different answers to the inflicted pain, such as tissue pressure, surface placing, and the handling frequency through the clinical research personnel that needed to be taken into account [64]. The work of the Hardy group to quantify pain thresholds and to design a scale based on Fechner’s concept of “just noticeable differences” (“*Unerschiedsschwelle*”) was at the frontier of contemporary neurophysiological pain research. It reinstated the psychophysiological conviction (as put forward earlier by Wundt, Weber, and Fechner) that a mathematical law could be found that established a direct connection between pain stimuli and patients’ feelings of being in pain and distress. While both clinical pain measurement practices and the handling of dolorimeter variants had their limitations, it had particularly been the rise of the psychopharmaceutical industry since the 1950s that led to new advances in algesiogenic pathology from both a diagnostic and therapeutic perspective. Moreover, with the parallel rise of intensive care medicine and the visible demographic shift, clinical neurologists, psychiatrists, and anesthesiologists increasingly dealt with ever-larger patient populations and new pain-related diseases [23].

## 4. Gate Control Theory

Since the second half of the twentieth century, several anesthesiologists, like the Italian-American physician John Bonica (1917–1994), joined neurological researchers, for example Willem Noordenbos (1875–1954) at the University of Amsterdam and Patrick David Wall (1925–2001) at University College London, along with experimental psychologists, such as Donald Hebb and Ronald Melzack at Montreal’s McGill University, in order to craft new lines of investigation in experimental research settings. They were determined to find ways of arriving at a procedural concept of pain as both a complex and subjective entity. This process went hand in hand with the creation of experimental pain research centers, in which patients and physicians attempted to understand and treat pain in more specific forms. Shortly after the Second World War (and largely triggered through the experiences from psychiatric and medical treatments of veteran soldiers), the specific mechanisms still remained unknown. This included differences in subjective suffering from similar injuries; pathological changes aligned with drastic pain symptoms in one person, but not in another person; forms of phantom pain; headaches without somatic symptomatologies; and the ever-lasting issue of how placebos and suggestive therapies likewise led to successful pain relief [65].

The physicians of the time worked hard on developing new answers to understanding and investigating pain phenomena, such as the founding figure of modern pain research Henry K. Beecher. Beecher had been a Kansas-born anesthesiologist, who took his medical training at Harvard and graduated from medical school in 1932. Twelve years later, Beecher had joined the medical staff at the Northern Italian campaign of Anzio, where Allied and Axis forces endured ferocious battles with many casualties during the year of 1944. As a consequence, approximately 7,000 American and British troops had died, 36,000 troops were either wounded or missing in action, and a concerning number of 44,000 were treated by psychiatrists and neurologists with forms of non-battle related illness. In his practice as a military field doctor, Beecher not only cared for soldiers’ somatic wounds, fractures, and diseases, but tried to alleviate their resulting pain conditions as well. He and his military medical colleagues thereby noticed that the gravity of a soldier’s injury seldom represented their behaviours in seeking active pain relief. This phenomenon did not square with current-day theories about pain development or anesthesiological treatment, which (following the lines of psychophysiological testing since the latter half of the nineteenth century) rather saw a direct relationship between a physical condition and the psychological perception. When he had returned to the United States, Beecher devoted his medical research career almost completely to the understanding of this phenomenon, while trying to create an intriguing theory for the conceptualization of pain that could accommodate for the striking symptomatologies he had seen at Anzio in his earlier medical practice. He thus arrived at a new apprehension of the social meanings of pain, as these (subjectively) affected persons’ interactions with pain and the respective treatment options [66].

There is hardly any doubt that Beecher’s investigations exerted an extraordinary influence on other postwar research groups that grappled with the problem of an objective identification of the subjective dimension of pain processes in experimental and clinical settings. The development of the MPQ itself represented a major milestone along this trajectory, in that it offered “quantitative measures of clinical pain that can be treated statistically.” McGill-based experimental psychologist Melzack and neuroanatomist Patrick Wall presented their influential “gate control theory” of pain already in 1965, yet it was their later publication of the MPQ that led to a watershed moment in the clinical assessment of pain since the mid-1970s. The Montreal group’s evidential basis had been formed by nearly three hundred patients, who suffered from pain in cancer, phantom limbs, obstetric conditions, or as a result of previous surgery, *etc*. Melzack had collected the actual words and linguistic phrases that contemporary patients used to characterize their conditions. The MPQ, when it became available, was so successful that it remained one of the most widespread methods to assess pain globally until more refined test protocols became available during the 1990s [67]. The MPQ was furthermore translated into more than sixty languages and has fared as a methodological basis for half-a-thousand pain-related clinical and psychological studies since [68].

Even recently, in 2012, American physician David Biro referred to the MPQ as perhaps the greatest effort in the long history of pain relief to assist patients in identifying and characterizing their pain conditions. It is striking however that the MPQ, as much as many other pain questionnaires and rating scales, has been confined to the use in specialized pain clinics rather than emerging as a tool for other medical professions or family doctors. While it offered patients an opportunity to describe their subjective perceptions and feelings, according to Biro’s account, general physicians, who were themselves trained in the scientific and objectifying experimental tradition, remained largely “uncomfortable with the form’s metaphorical language” along with their patients insistence on a the use of a more accessible language for their conditions, to which somatic physicians were not yet attuned to [69]. This is of course a quite paradoxical finding, since the MPQ had itself been designed to capture patients’ subjective pain experience through the use of innovative linguistic means in a scientific and objectifying attempt. Therefrom resulted an integrative notion of pain as a complex, personal, and changing entity, which allowed physicians to understand their patients’ pain experience better, much like Fechner and Weber had stipulated in their earlier psychophysiological accounts during the mid- nineteenth century [40].

## 5. Aftermath

With respect to the historiographical considerations of the research and visualization practices used by neurophysiologists during the nineteenth and clinicians during the twentieth century in their endeavours to objectify “pain” phenomena, one may recognize an increasing fascination of contemporary neuroscientists with imaging techniques. Visualizations of physiological pain processes became thereby paired with contemporary scientific and aesthetic perspectives [70]. In this respect, California-based historian of science Lisa Cartwright identified a fundamental, two-fold historiographical problem field. Owing to our current preoccupation with neuroimaging studies, which have even become synonymous with many trajectories of neuroscience, including clinical and basic pain research, its prehistory before the introduction of CT scanning around 1968 has almost fallen into oblivion [71]. In fact, this important historical research area is still considerably understudied, while the scientific presuppositions, methodological traditions, and epistemological problems have not been sufficiently explored (including the use of diagrammatic rating scales and earlier visualization approaches in pain research, such as electroencephalography in specialized clinics) in their changing social and cultural contexts [72]. In addition to being a fashionable eye-catching term, “neuroimaging” tendencies have also been used to carve out new funding niches and research collaborations in current-day neuroscience. In fact, the interdisciplinary character of neuroscientific pain research (involving clinicians, neurophysiologists, experimental psychologists, and neuroimaging specialists) has also to take the aesthetic, practical, and methodological foundations of medical imagery into account, particularly since important epistemological aspects of subjectivity *vs.* objectivity of pain, individual experience *vs.* inter-subjective meanings, along with cross-cultural comparisons of pain perceptions) are at stake. The relationship between this research tradition in the life sciences and multiple elements of laboratory practices and methodologies is quite under-represented with respect to epistemological questions of scientific innovation and the acknowledgement of respective achievements (as emanates from the constrained use of the MPQ questionnaire in specialized pain clinics *vis-à-vis* an insufficient uptake among general practitioners). Some historiographical case studies from the second half of the nineteenth century and beginning of the twentieth century have examined selected non-discursive practices of neurophysiological and neuroanatomical imaging. The relevant publications could hereby show that the research traditions evolved primarily from a collection- and comparison-directed activity to manufacture-based and, eventually, industrial endeavors. With regard to the development of pain theories, this evolution witnessed a vast increase in the pharmaceutical industry since the second half of the twentieth century [73]. It is further reflected in the history of magnetic resonance imaging (MRI), computed tomography (CT), and positron emission tomography (PET), which paved the way to a new paradigm of computer-aided medical visualization. At the same time, it should not go unnoticed that this development was certainly a non-linear one; so, for example, the advent of MRI re-defined what the collection of comparison-oriented brain activities could mean, while also influencing the subsequent introduction of PET-scanning technologies in pain research [74].

The genetic link between the objectification of pain in earlier nineteenth-century studies, especially those conducted by the Leipzig school of psychophysiology, continued to influence later research trends right into the post-World War Two period. This development can be noticed, for example, in several neuroimaging cases such as MRI [75], fMRI [76], PET-scanning [77], the visual human project^®^ [78] or applications in 3D gene regulation analyses [79]. The digital nature of the resulting images is now an essential feature of all of these visualization approaches. These technologies are information-based processes that frequently transcend the influence and reach of human perception and pain pathologies. In the computerized reprocessing of the information (such as in graphs, logarithmic abstractions, enhanced PET scans, *etc.*), much of the normal documentation of practical human endeavors, involving comforting and retraction behaviors in pain conditions, as well as written linguistic descriptions of pain-related experiences get transformed and “objectively” presented in aesthetic ways, which often render them “understandable” only to the experts’ eyes (such as the “lightening up” of the nucleus accumbens in an fMRI done with a patient in acute pain). This is often the result of dedicated interdisciplinary work by larger teams of neuroscientists, electronic designers, bioinformaticians and physical scientists, which has also led to the development of new interdisciplinary forms of reasoning, epistemic approaches, and forms of neuroscientific experimentation [80]. As such, we have come to witness an important co-evolution in the relationship between the life sciences and visualization techniques, as these had previously contributed to the investigative process of studying pain since the early 1980s.

Furthermore, the technological equipment of the laboratories itself has altered our understanding of the visualization of pain investigations, such as the preference for Siemens Scanners^®^ or the neurophysiological equipment of Kopf^®^. Such “minor details” do matter, in fact, since these offer specific features, which other forms of equipment generally would entail. These practical and methodological choices accordingly permit different perceptions about the experimental objects or may lead to altered plots and printouts of results to be published in biomedical journals [81]:

“It is often thought that there is a unique and specific kind of barrier which protects science, and particularly scientific judgment, against the intrusion of so-called ‘external influences’, and that the barrier fails only in highly exceptional and unusual circumstances which can properly be considered pathological... Certainly, the evidence presently available suggests that “external” influences upon scientific judgment are neither unusual nor necessarily pathological, and that the barrier, which such influences have to penetrate, is not fundamentally different from the boundaries surrounding other sub-cultures.”

[82]

If we do include such instances within the general, and thus accepted, visual, psychophysiological and aesthetic repertoires of modern neuroscientists, then we could open up exciting venues and intriguing fields of research. This may also help to lower the gap between the specific methods of a modern algesiogenic science of pain and its grappling with the objectifying tradition since the nineteenth century. Potentially, the raising of an awareness about the subjective side of interpretations at both ends, the patients’ distress and the researchers’ observations, would help in carving out an important middle ground for the semi-objective or inter-subjective understanding of the “pain dimension.”

In accordance with the view that subjective judgments, human perceptions, and aesthetic considerations have all become important factors in contemporary knowledge processes, such elements have likewise been reintroduced into the scholarly analysis of the modern biomedical sciences [83]. Even beyond the activities of individual researchers, the laboratory-centered technological arrangements display the aesthetic values and judgments of the respective researchers that determine the future research practices in pain neuroscience as well [84]. These experimental arrangements have come to unfold a contingent form of “laboratory life,” similar to Johannes Mueller’s preference to experiment with young puppies (which he could easily get from the street markets of Berlin), his frequent use of pinfeathers in the experiments (as these became recycled in related experiments), or the applicability of chemical substances (such as the available “Prussian blue” stains). At the same time, Mueller lacked access to quantifying measurement devices or microphotographic equipment for his pain research activities [85]. One may see these instances nevertheless as clear examples of subjective and aesthetic preference decisions, which has also been pointed out in recent anthropological studies focussing on subjective articulations of pain [86]. Thus, issues of uncertainty and randomness prevail in today’s biomedical pain research approaches; problem areas that nineteenth-century physiologists were quite aware of, due to their constant debates with clinicians over the status of the research findings in their neurophysiological laboratories [87].

## 6. Discussion of “Algesiogenic Pathology”

The American journalists and authors Nancy Campbell, J. P. Olsen and Luke Walden have directed both the public’s and scholars’ attention to the development of a transformed “world of pain.” In their coauthored book “*The Narcotic Farm: The Rise and Fall of America’s First Prison for Drug Addicts*” they have contextualized the rising phenomenon of addiction in medicine and psychiatry in the U.S. within the development of more and more autonomous pain clinics in the healthcare system [88]. Campbell and her coauthors placed this new trend towards an algesiogenic pathology also in the general evolution of the demographic change and an increase in the subject of chronic illness and disease in the Western word, which has led to more and more expert chronic pain diagnoses by physicians [89]. Through this emergence of chronic pain as an autonomous medical and cultural object, also an augmented notion of “pain” as a subjective though in principle measurable unit came to the fore. At the same time, however, certain pain phenomena (e.g., thalamic pain, certain headache forms, oncological pain syndromes, *etc.*) also resisted the new test paradigms as well as the pharmacological treatments. This happened despite the circumstance that during the twentieth century, the issue of “chronic pain” halted the traditional understanding of a predominantly subjective approach to pain in medicine and psychiatry, while ever more specialized treatment options, pain management approaches, as well as clinical and addiction institutions were opened. One can find these persistent problems in pain management in the further development of the Montreal MPQ or in the continued investigations of the New York program around Hardy and his co-workers, while they strove to include subjective approaches into objective pain measurement scales. Although the pain research groups in Montreal and New York had worked with different research practices, which included, for example, the self-descriptions in a linguistic repertoire or the application of a specialized dolorimeter, while continuing to develop robust measurement paradigms. These paradigms sought to represent a multitude of patient histories and pain processes over time. Both had their respective strengths and weaknesses. The Montreal approach, for example, prioritized the subjective accounts of pain, while the New York protocol strove to include contextual influences on varying pain perceptions in the first place. As a result, the pain questionnaires and dolorimetric approaches have continued treating pain as a measurable psychophysiological phenomenon, while developing assessment protocols that incorporated somatic markers and personal histories in the measurement processes as well [90].

The investigative approaches that Melzack and Torgerson pursued in Canada and Hardy’s group in the United States could also be interpreted as specifically semi-subjective measurements, as described as a consistent development in the general argument of this article. The experimental physiologists of the nineteenth century, particularly Weber and Fechner from the Leipzig school of psychophysiology, proposed a particular and laboratory-based investigation of pain processes that led to an increasing objectification in pain research. The German researchers, respectively, based their paradigm on individual experimental concepts of “thresholds” and “normal values” in their laboratory settings. However, starting with the aftermath of the Second World War, pain research got to include a complex process of neuronal signal computation, brain plasticity, and neuroimaging in ever more sophisticated programs of investigation. The Montreal group identified particularly the 1950s as a watershed in pain research, since the relevant central nervous pathways had now been singled out in their entirety. Their hope for an increasingly detailed somatic understanding of pain processes was however strongly augmented by Hardy’s co-workers at Cornell, who could demonstrate a persistent unevenness in the perception of experimental and medical pain conditions, as well as among groups of people with noticeable cultural differences [91].

## 7. Conclusions

The increasing issue of “chronic pain” has turned to include the historical nineteenth-century approach to an experimental objectification of pain together with postwar developments towards semi-subjective pain questionnaires and rating scales, which emerged as the “gold standard” in algesiogenic pathological research. Yet, apart from being used to develop medical funding niches within the modern field of neuroscience itself, with its interdisciplinary research character, additional epistemological problems surfaced from practical, often aesthetic, cultural and methodological investigations. The relationship between the life sciences and multiple components of research practices are still a widely under-researched area―when the epistemological subjects of “psychogenic pain” or the “pain-prone patient” are taken into account [92]. Some historical case studies regarding the second half of the nineteenth and beginning of the twentieth century have hitherto investigated the impact of the social and instrument-based practices in neurophysiology and neuroanatomy. These studies could show that the laboratory research practices often progressed from a primarily comparison-oriented activity, regarding the emergence of experimental physiology that laid the foundations for later twentieth-century developments in the neurosciences, to an ever more practical and investigative scientific enterprise. The experimental and visual representations, along with the interrelation of textual―and often diagrammatic (in the case of pain questionnaires and research scales)―structures with visual products, have become the subject of considerable historical research over the past fifteen years. The “iconic turn” and investigation of “visual cultures” in the history of science have thereby moved in a direction of ever-deeper analyses of the media products (objects, graphs, histological sections, neuroimages, *etc.*) in the life sciences, where the field of an algesiogenic pathology has not been an exception, but another important instance of study [93]. The current article has underlined the methodological and experimental advances in the wider field of neurophysiological and clinical pain research, together with the historical emergence of related imaging practices in the modern neurosciences [94]. It has further mapped some important developments from the mid-nineteenth century to the later twentieth century, in which patients with pain conditions have become the subjects of a new neurological “way of seeing;” it now combined the visualization of pain with the disciplining of patients through objectifying phenomenologies and physiologies within the modern interdisciplinary research settings.
